# Urolithiasis Is a Risk Factor for Uroseptic Shock and Acute Kidney Injury in Patients With Urinary Tract Infection

**DOI:** 10.3389/fmed.2019.00288

**Published:** 2019-12-05

**Authors:** Chih-Yen Hsiao, Tsung-Hsien Chen, Yi-Chien Lee, Meng-Chang Hsiao, Peir-Haur Hung, Yih-Yuan Chen, Ming-Cheng Wang

**Affiliations:** ^1^Division of Nephrology, Department of Internal Medicine, Ditmanson Medical Foundation Chia-Yi Christian Hospital, Chiayi, Taiwan; ^2^Department of Hospital and Health Care Administration, Chia Nan University of Pharmacy and Science, Tainan, Taiwan; ^3^Department of Internal Medicine, Fu Jen Catholic University Hospital, Fu Jen Catholic University, New Taipei City, Taiwan; ^4^School of Medicine, College of Medicine, Fu Jen Catholic University, New Taipei City, Taiwan; ^5^The Jackson Laboratory for Genomic Medicine, Farmington, CT, United States; ^6^Department of Applied Life Science and Health, Chia Nan University of Pharmacy and Science, Tainan, Taiwan; ^7^Department of Biochemical Science and Technology, National Chiayi University, Chiayi, Taiwan; ^8^Division of Nephrology, Department of Internal Medicine, National Cheng Kung University Hospital, College of Medicine, National Cheng Kung University, Tainan, Taiwan

**Keywords:** urolithiasis, urinary tract infection, uroseptic shock, acute kidney injury, bacteremia

## Abstract

Urinary tract infection (UTI) is a common complication in patients with urolithiasis. This study aimed to compare clinical manifestations and treatment outcomes among UTI patients with or without urolithiasis. It also focused on identifying relationships among urolithiasis, uroseptic shock, and acute kidney injury (AKI). This retrospective study enrolled hospitalized UTI patients who underwent imaging in an acute care setting from January 2006 to March 2015. Of 662 participants enrolled, 113 (17.1%) had urolithiasis, 107 (16.2%) developed uroseptic shock, and 184 (27.8%) developed AKI. A multivariate logistic regression analysis showed that in UTI patients, urolithiasis is associated with an increased risk of uroseptic shock (OR 1.80, 95% CI: 1.08–3.02, *P* = 0.025), AKI (OR 1.95, 95% CI: 1.22–3.12, *P* = 0.005), and bacteremia (OR 1.68, 95% CI: 1.08–2.64, *P* = 0.022). Urolithiasis is common in UTI patients and is associated with an increased risk of uroseptic shock and AKI.

## Introduction

Urolithiasis is a global problem with an increasing incidence and prevalence rate (1, 2). The global prevalence of urolithiasis ranges from 2 to 20% and varies based on geographical locations and socioeconomic conditions of different populations (3, 4). Urolithiasis is a well-known risk factor of urinary tract infection (UTI), and a vicious cycle leading to several clinical consequences is observed seen as follows: stones → obstruction → stasis → infection → stones (5). Furthermore, urolithiasis is associated with an increased risk of chronic kidney disease (CKD), hypertension, and myocardial infarction (6–8).

Urolithiasis can lead to urinary stasis, which enables bacteria to adhere to the urothelium and multiply, thereby causing UTI (9). Complicated UTI related to obstructive uropathy can progress to urosepsis and may cause septic shock and disseminated intravascular coagulopathy (10). However, only a few studies have demonstrated the correlation among urolithiasis, uroseptic shock, and acute kidney injury (AKI) in UTI patients. Hence, we conducted a study to investigate whether urolithiasis is an important risk factor for uroseptic shock and AKI in UTI patients.

## Materials and Methods

### Study Design and Patient Selection

The data for this retrospective observational study were collected between January 2006 and March 2015 from patients visiting Chiayi Christian Hospital in southern Taiwan, which consists of 1,077 inpatient beds and an outpatient department serving ~4,110 patients per day. This study was conducted after obtaining ethical approval from the Institutional Review Board of Chiayi Christian Hospital (approval no. CYCH-IRB-100015).

In total, 662 consecutively hospitalized UTI patients without any other concurrent infectious disease were enrolled in the study. All patients were adults and presented with UTI symptoms such as pain on urination, lumbago, or fever with bacterial isolation of >10^5^ colony-forming units/mL from a urine specimen. All patients underwent imaging studies, such as ultrasonography, intravenous urography (IVU), or computed tomography (CT), to identify urolithiasis and urinary tract obstruction. The patients were divided into two groups based on the presence or absence of urolithiasis. The clinical characteristics and laboratory data were collected using a standard form for further analysis.

### Assessment of the Subjects

Data on demographic characteristics (age and sex), comorbidities [diabetes mellitus (DM), hypertension, coronary artery disease, congestive heart failure (CHF), stroke, and long-term indwelling Foley catheter], vital signs (blood pressure and temperature), laboratory results [white blood cell (WBC) count, platelet count, serum creatinine, and estimated glomerular filtration rate (eGFR) at baseline and after hospitalization], causative microorganisms and antimicrobial resistance pattern, and imaging of patients were collected and analyzed.

### Definitions

The diagnosis of urolithiasis was based on imaging studies. Urolithiasis was defined as the presence of any stone formation in the urinary tract that was detected during imaging studies such as ultrasonography, CT with or without enhancement, or IVU. Uroseptic shock was defined as sepsis-induced hypotension [systolic blood pressure (SBP) <90 mmHg or mean arterial pressure <70 mmHg or a decrease in SBP by >40 mmHg and lasting for at least 1 h, despite adequate fluid resuscitation and in the absence of other causes of hypotension] (11). eGFR was determined using the Chronic Kidney Disease Epidemiology Collaboration (CKD-EPI) creatinine equation (12, 13). For patients without recognized CKD, the estimation of baseline creatinine level was performed using the CKD-EPI creatinine equation assuming a GFR of 75 ml/min/1.73 m^2^ (14). AKI was defined as an increase in serum creatinine level to ≥1.5 times the baseline values according to the KDIGO Clinical Practice Guideline criteria for serum creatinine value for AKI stages 1, 2, and 3 (15). Afebrile status was defined as a body temperature of ≤ 38.3°C (101°F) during hospitalization. Multiple drug resistance (MDR) was defined as non-susceptibility of the isolates to at least one agent in three or more antimicrobial categories (16).

### Statistical Analyses

The patients were grouped into two categories: those with or without urolithiasis. The continuous variables were expressed as mean ± SD, and the categorical variables were expressed as number (percentage). The data were analyzed using Student's *t*-test or chi-square test depending on the type of variables. Multivariate logistic regression analyses were performed to identify factors associated with urolithiasis, septic shock, and AKI post-admission. Only variables significant at the 0.15 level in the univariate analysis were selected for consecutive multivariate analyses. The goodness-of-fit of the logistic regression model was assessed using the Cox and Snell test, and the explanatory power was reported using Nagelkerke's pseudo-R-square. A *P*-value of <0.05 was considered statistically significant. All analyses were performed using SPSS software for Windows (SPSS Science, v. 17.0, Chicago, IL).

## Results

The demographic and clinical characteristics of 662 hospitalized UTI patients are shown in Table 1. The mean age on admission was 67 ± 17 years. A majority of the patients [468 (70.7%)] were females, and 229 (34.6%) had a prior history of UTI. There were 107 patients (16.2%) with uroseptic shock and 184 patients (27.8%) with AKI during hospitalization. The overall mortality rate was 0.45% (3/662). Among UTI patients, 113 (17.1%) had urolithiasis and 41 had ureteral stone with urinary tract obstruction. The ureteral stones were located at the upper ureter or ureteropelvic junction in 18 patients (43.9%), mid ureter in 5 (12.2%), and distal ureter and ureterovesical junction in 18 (43.9%). Thirty (16.3%) and 81 patients (16.9%) underwent radiologic examinations using contrast media (IVU or CT) in the AKI and non-AKI groups, respectively. The prevalence of male sex (40.7 vs. 27.0%, *P* = 0.003), bacteremia (57.5 vs. 40.3%, *P* = 0.001), uroseptic shock (26.5 vs. 14.0%, *P* = 0.001), AKI (40.7 vs. 25.1%, *P* = 0.001) and *Proteus* spp. isolates (10.6 vs. 2.6%, *P* < 0.001) was higher, while *Escherichia coli* isolates (61.1 vs. 75.8%, *P* = 0.002) was lower in patients with urolithiasis than in those without. Among UTI patients with urolithiasis, the incidences of AKI were 60.0 and 33.7%, respectively, in patients with and without uroseptic shock. The top five bacteria identified in UTI patients with urolithiasis were *E. coli* (61.1%), *Proteus* spp. (10.6%), *Klebsiella* spp. (8.8%), *Pseudomonas* spp. (8.0%), and *Enterococcus* spp. (3.5%). The top five bacteria identified in UTI patients without urolithiasis were *E. coli* (75.8%), *Klebsiella* spp. (7.7%), *Pseudomonas* spp. (7.1%), *Enterococcus* spp. (4.6%), and *Proteus* spp. (2.6%).

**Table 1:** Characteristics of hospitalized patients with urinary tract infection with respect to urolithiasis.

	**All (*n* = 662)**	**Non-urolithiasis (*n* = 549)**	**Urolithiasis (*n* = 113)**	***P*-value**
Age (year)	67 ± 17	67 ± 18	67 ± 15	0.603^*^
Sex (male)	194 (29.3)	148 (27.0)	46 (40.7)	0.003^¥^
Diabetes mellitus	282 (42.6)	232 (42.3)	50 (44.2)	0.697^¥^
Hypertension	335 (50.6)	275 (50.1)	60 (53.1)	0.561^¥^
Congestive heart failure	28 (4.2)	23 (4.2)	5 (4.4)	0.802^¥^
Coronary artery disease	64 (9.7)	55 (10.0)	9 (8.0)	0.501^¥^
Stroke	157 (23.7)	134 (24.4)	23 (20.4)	0.356^¥^
Prior history of UTI				0.726^¥^
None	433 (65.4)	357 (65.0)	76 (67.3)	
Once	123 (18.6)	105 (19.1)	18 (15.9)	
Twice	59 (8.9)	50 (9.1)	9 (8.0)	
Thrice or more	47 (7.1)	37 (6.7)	10 (8.8)	
Indwelling Foley catheter	58 (8.8)	46 (8.4)	12 (10.6)	0.443^¥^
Afebrile	270 (40.8)	231 (42.1)	39 (34.5)	0.136^¥^
Bacteremia	286 (43.2)	221 (40.3)	65 (57.5)	0.001^¥^
Uroseptic shock	107 (16.2)	77 (14.0)	30 (26.5)	0.001^¥^
Acute kidney injury	184 (27.8)	138 (25.1)	46 (40.7)	0.001^¥^
Acute kidney injury stage				0.004^¥^
Stage 1	99 (15.0)	78 (14.2)	21 (18.6)	
Stage 2	58 (8.8)	41 (7.5)	17 (15.0)	
Stage 3	27 (4.1)	19 (3.5)	8 (7.1)	
Baseline serum creatinine (mg/dL)	1.1 ± 0.8	1.1 ± 0.8	1.1 ± 0.8	0.665^*^
Baseline eGFR (mL/min/1.73 m^2^)	70.5 ± 28.7	70.4 ± 29.7	71.2 ± 23.6	0.755^*^
Hospitalized serum creatinine (mg/dL)	1.6 ± 1.4	1.6 ± 1.4	1.7 ± 1.4	0.561^*^
Hospitalized eGFR (mL/min/1.73 m^2^)	58.2 ± 36.4	56.0 ± 32.2	50.9 ± 27.1	0.076^*^
White blood cell (10^3^/μL)	13.4 ± 6.1	13.4 ± 5.9	13.7 ± 7.3	0.649^*^
Platelets (10^3^/μL)	207 ± 124	209 ± 130	202 ± 92	0.600^*^
*Escherichia coli*	485 (73.3)	416 (75.8)	69 (61.1)	0.002^¥^
*Proteus* species	26 (3.9)	14 (2.6)	12 (10.6)	<0.001^¥^
*Klebsiella* species	52 (7.9)	42 (7.7)	10 (8.8)	0.811^¥^
*Enterococcus* species	29 (4.4)	25 (4.6)	4 (3.5)	0.803^¥^
*Pseudomonas* species	48 (7.3)	39 (7.1)	9 (8.0)	0.903^¥^
MDR isolate	234 (35.3)	190 (34.6)	44 (38.9)	0.381^¥^

* Student's t-test;

¥ *Chi-square test or Fisher's exact test*.

*Data are expressed as mean ± SD or number (percentage)*.

*MDR, multiple drug resistance; eGFR, estimated glomerular filtration rate*.

**Figure 1:**
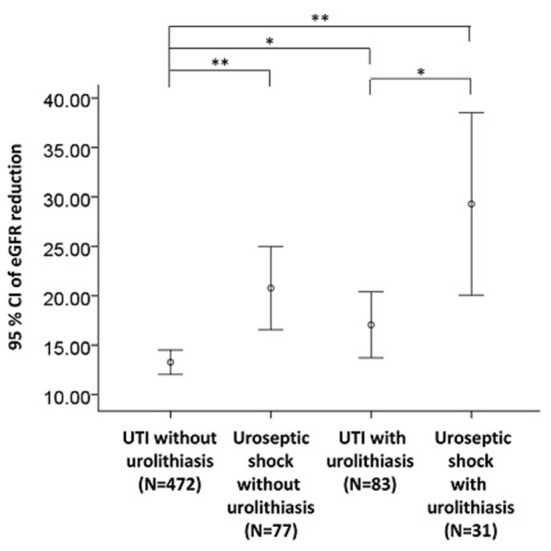
eGFR reduction in UTI patients with respect to uroseptic shock and urolithiasis. eGFR, estimated glomerular filtration rate; UTI, urinary tract infection; eGFR reduction, baseline minus worst value of eGFR. *p < 0.05, **p < 0.01.

CHF (OR 2.43, 95% CI: 1.03–5.77, *P* = 0.043), urolithiasis (OR 1.80, 95% CI: 1.08–3.02, *P* = 0.025), and AKI (OR 2.30, 95% CI: 1.45–3.65, *P* < 0.001) were independently associated with an increased risk of uroseptic shock (Table 2). Patients with DM (OR 1.78, 95% CI: 1.21–2.60, *P* = 0.003), afebrile during hospitalization (OR 1.63, 95% CI: 1.08–2.44, *P* = 0.019), bacteremia (OR 2.19, 95% CI: 1.47–3.26, *P* < 0.001), uroseptic shock (OR 2.44, 95% CI: 1.51–3.93, *P* < 0.001), urolithiasis (OR 1.95, 95% CI: 1.22–3.12, *P* = 0.005), and higher WBC count (OR 1.04, 95% CI: 1.00–1.07, *P* = 0.025) were independently associated with an increased risk of AKI. Conversely, higher eGFR (OR 0.99, 95% CI: 0.98–0.99, *P* < 0.001) was independently associated with a decreased risk of AKI in UTI patients (Table 3). Figure 1 shows a reduction in eGFR in UTI patients with uroseptic shock and urolithiasis. The reduction in eGFR values in uroseptic shock patients with or without urolithiasis, and UTI patients with or without urolithiasis were 28.35 (95% CI: 19.23–37.46), 20.78 (95% CI: 16.58–24.97), 17.05 (95% CI: 13.72–20.39), and 13.28 (95% CI: 12.05–14.50) mL/min/1.73 m^2^, respectively. Significant differences were observed between uroseptic shock patients with urolithiasis and UTI patients with urolithiasis, uroseptic shock patients without urolithiasis and UTI patients without urolithiasis, uroseptic patients with urolithiasis and UTI patients without urolithiasis, and UTI patients with urolithiasis and UTI patients without urolithiasis. Urolithiasis (OR 1.68, 95% CI: 1.08–2.64, *P* = 0.022), AKI (OR 1.87, 95% CI: 1.26–2.78, *P* = 0.002), and higher WBC count (OR 1.04, 95% CI: 1.01–1.07, *P* = 0.006) were independently associated with an increased risk of bacteremia, while afebrile status (OR 0.32, 95% CI: 0.22–0.46, *P* < 0.001) and higher platelet count (OR 1.00, 95% CI: 0.99–1.00, *P* < 0.001) were independently associated with a decreased risk of bacteremia in UTI patients (Table 4).

**Table 2:** Univariate and multivariate logistic regression analyses of factors related to uroseptic shock in patients with urinary tract infection.

**Covariate**	**Univariate**		**Multivariate**	
	**OR (95% CI)**	***P*-value**	**OR (95% CI)**	***P*-value**
Age (year)	1.01 (1.00–1.03)	0.038	1.01 (0.99–1.02)	0.230
Sex (male)	1.48 (0.96–2.29)	0.077	1.49 (0.94–2.37)	0.093
Diabetes mellitus	0.93 (0.61–1.42)	0.736		
Hypertension	0.83 (0.55–1.26)	0.382		
Congestive heart failure	3.08 (1.38–6.86)	0.006	2.43 (1.03–5.77)	0.043
Coronary artery disease	2.04 (1.12–3.71)	0.019	1.54 (0.80–2.94)	0.195
Stroke	1.31 (0.82–2.09)	0.252		
Indwelling Foley catheter	0.95 (0.45–1.99)	0.889		
Afebrile	0.66 (0.43–1.03)	0.065	0.62 (0.38–1.02)	0.060
Bacteremia	1.95 (1.28–2.96)	0.002	1.34 (0.84–2.15)	0.216
Acute kidney injury	2.76 (1.80–4.22)	<0.001	2.30 (1.45–3.65)	<0.001
Urolithiasis	2.22 (1.37–3.59)	0.001	1.80 (1.08–3.02)	0.025
Baseline eGFR (mL/min/1.73 m^2^)	1.00 (0.99–1.00)	0.292		
White blood cell (10^3^/μL)	1.02 (0.99–1.06)	0.157		
Platelets (10^3^/μL)	1.00 (1.00–1.00)	0.024	1.00 (1.00–1.00)	0.166

*eGFR, estimated glomerular filtration rate*.

**Table 3:** Univariate and multivariate logistic regression analyses of factors related to acute kidney injury in patients with urinary tract infection.

**Covariate**	**Univariate**		**Multivariate**	
	**OR (95% CI)**	***P*-value**	**OR (95% CI)**	***P*-value**
Age (year)	1.03 (1.02–1.04)	<0.001	1.01 (0.99–1.03)	0.206
Sex (male)	0.80 (0.55–1.18)	0.260		
Diabetes mellitus	2.19 (1.55–3.10)	<0.001	1.78 (1.21–2.60)	0.003
Hypertension	1.95 (1.38–2.77)	<0.001	1.30 (0.87–1.95)	0.197
Congestive heart failure	2.73 (1.27–5.84)	0.010	1.84 (0.78–4.32)	0.161
Coronary artery disease	1.77 (1.04–3.02)	0.036	1.13 (0.62–2.05)	0.698
Stroke	1.02 (0.68–1.51)	0.941		
Indwelling Foley catheter	0.99 (0.54–1.81)	0.970		
Afebrile	1.45 (1.03–2.04)	0.035	1.63 (1.08–2.44)	0.019
Bacteremia	2.11 (1.50–2.99)	<0.001	2.19 (1.47–3.26)	<0.001
Uroseptic shock	2.76 (1.80–4.22)	<0.001	2.44 (1.51–3.93)	<0.001
Urolithiasis	2.04 (1.34–3.12)	0.001	1.95 (1.22–3.12)	0.005
Baseline eGFR (mL/min/1.73 m^2^)	0.98 (0.98–0.99)	<0.001	0.99 (0.98–0.99)	<0.001
White blood cell (10^3^/μL)	1.04 (1.01–1.07)	0.004	1.04 (1.00–1.07)	0.025
Platelets (10^3^/μL)	1.00 (1.00–1.00)	0.568		

*eGFR, estimated glomerular filtration rate*.

**Table 4:** Univariate and multivariate logistic regression analyses of factors related to bacteremia in patients with urinary tract infection.

**Covariate**	**Univariate**		**Multivariate**	
	**OR (95% CI)**	***P*-value**	**OR (95% CI)**	***P*-value**
Age (year)	1.00 (0.99–1.01)	0.499		
Sex (male)	0.95 (0.68–1.33)	0.755		
Diabetes mellitus	1.32 (0.97–1.81)	0.077	1.30 (0.91–1.85)	0.146
Hypertension	1.26 (0.92–1.71)	0.146	1.41 (0.99–2.00)	0.059
Congestive heart failure	0.99 (0.46–2.12)	0.970		
Coronary artery disease	1.18 (0.70–1.98)	0.533		
Stroke	0.66 (0.46–0.96)	0.030	0.70 (0.46–1.06)	0.088
Indwelling Foley catheter	0.73 (0.42–1.27)	0.262		
Afebrile	0.34 (0.24–0.47)	<0.001	0.32 (0.22–0.46)	<0.001
Uroseptic shock	1.95 (1.28–2.96)	0.002	1.42 (0.89–2.26)	0.138
Acute kidney injury	2.11 (1.50–2.99)	<0.001	1.87 (1.26–2.78)	0.002
Urolithiasis	2.01 (1.33–3.03)	0.001	1.68 (1.08–2.64)	0.022
Baseline eGFR (mL/min/1.73 m^2^)	1.00 (0.99–1.00)	0.519		
White blood cell (10^3^/μL)	1.03 (1.01–1.06)	0.017	1.04 (1.01–1.07)	0.006
Platelets (10^3^/μL)	1.00 (1.00–1.00)	<0.001	1.00 (0.99–1.00)	<0.001

*eGFR, estimated glomerular filtration rate*.

## Discussion

The relationship between urolithiasis and UTI is complex. UTI is a common complication in patients with urolithiasis, and it promotes the formation of urolithiasis (5). Our study demonstrated a higher prevalence of urolithiasis in UTI patients than in the general population. The presence of urolithiasis was associated with worse clinical outcomes in UTI patients, including an increased risk of bacteremia, uroseptic shock, and AKI. To the best of our knowledge, this is the first study to demonstrate that urolithiasis is associated with an increased risk of uroseptic shock and AKI in UTI patients.

Patients with septic shock are in a critical situation that requires significant healthcare resources. It is estimated that UTI is the underlying cause in 30% of patients with severe sepsis and septic shock (17). The rate of developing septic shock in patients with UTI can range from 20.8 to 32.9% based on different underlying conditions (18–20). In the current study, the incidence of uroseptic shock in UTI patients that necessitated admission was 16.2%. Multiple factors attributing to septic shock have previously been reported in patients with UTI. Indwelling urinary catheter and increased C-reactive protein levels were risk factors of septic shock in UTI patients with or without bacteremia (18, 21). Our study showed that urolithiasis could be a good predictor of the development of uroseptic shock in UTI patients. Previous studies showed that the rate of uroseptic shock in calculous acute pyelonephritis (APN) ranged from 20.8 to 36.2% (22, 23). Here, we found a similar incidence (26.5%) of uroseptic shock in UTI patients with urolithiasis. UTI with urinary tract obstruction can lead to urosepsis in ~10% of cases (24), and the presence of urinary tract obstruction is a risk factor for septic shock in patients with bacteremic APN (19, 25). A lack of decompression leads to an increased risk of mortality for patients with sepsis and ureteral stone (26). It is important to decompress the renal collecting system by ureteral stent or pyelonephrostomy for UTI patients with severe urinary tract obstruction (27). Because urolithiasis is an independent risk factor for the development of septic shock in UTI patients, early imaging for sepsis patients with clinical suspicion of urinary source and decompressing the urinary collecting system for those with obstruction is recommended. For patients identifying severe sepsis or septic shock, administration of effective intravenous antibiotics within an hour is critical according to the recommendation of Surviving Sepsis Campaign guidelines (11).

The primary mechanism of urolithiasis-associated AKI is obstructive nephropathy (28). However, urolithiasis is a rare cause of adult AKI, accounting for ~1–2% of all AKI events only (28). One of the common causes of AKI in critically ill patients is sepsis (29), and ~60% of patients with septic shock develop AKI (30). Urolithiasis is a risk factor for UTI, and UTI is one of the common causes of sepsis, which may lead to uroseptic shock and deterioration of renal function. Our study found a high incidence of AKI (46.7%) among the patients with uroseptic shock, and uroseptic shock was an independent risk factor for AKI. UTI patients with urolithiasis were more prone to develop uroseptic shock and tended to have a greater reduction in eGFR than those without urolithiasis, regardless of the presence of uroseptic shock. Because patients with urolithiasis or uroseptic shock are both predisposed to develop more severe AKI, nephrotoxic agents, including non-steroidal anti-inflammatory drugs, contrast media, and aminoglycosides should be avoided in patients with a high risk of AKI.

The presence of bacteremia in complicated APN patients results in an increased risk of developing severe sepsis or uroseptic shock (31). Previous studies have indicated that specific clinical features in UTI patients, such as old age, low SBP, high body temperature, and high procalcitonin levels, were significantly associated with bacteremia (32). Our study showed that UTI patients with urolithiasis, AKI, and higher WBC count were at a higher risk of developing bacteremia. Urolithiasis *per se* is an important source of secondary infection (33). A previous study reported that the incidence of bacteria isolated from urine and stone matrices of stone formers was 24 and 32%, respectively (34). Traditionally, UTI with urease-producing bacteria, in most cases belonging to *Proteus* spp., can split urinary urea and increase urinary pH, thus, promoting the precipitation and aggregation of struvite crystals to form infective urolithiasis (5). However, UTIs are frequently associated with kidney stones of different chemical compositions other than struvite (34). Tavichakorntrakool et al. reported that the three most common bacteria in urine samples of patients with renal stones were *E. coli, Enterococcus* spp., and *Klebsiella*/*Enterobacter* spp. (34). Our data showed that the three most common pathogens in UTI patients with urolithiasis were *E. coli, Proteus* spp., and *Klebsiella* spp. There were more isolates of *Proteus* spp. but fewer isolates of *E. coli* in UTI patients with urolithiasis than in those without urolithiasis. Third-generation cephalosporin is a reasonable empirical antibiotic for stable community-acquired UTI associated with urolithiasis, while broader spectrum antimicrobial coverage is needed for patients with severe sepsis or septic shock (18). In addition, urolithiasis contains bacteria, and the number of bacteria on the stone surface can increase despite antibiotic therapy. Thus, eradication of the associated UTI is only possible after complete removal of the stone (35).

Our study had several limitations. First, the retrospective design involved a potential bias in data collection. Although we collected data using a standard form to reduce bias, the data regarding prior history of urolithiasis, as well as the type and size of urolithiasis were absent. A prospective study is needed to collect the above information to determine the associations between urolithiasis burden and urosepsis, as well as uroseptic shock and AKI. Second, this was a single-center study involving hospitalized patients with UTI, which limits the generalizability of the results. A multicenter study with a larger sample size will be needed to confirm our results. Third, in this study, the patient number with urinary tract obstruction was insufficient to evaluate it as a risk factor for causing uroseptic shock and AKI in UTI patients with urolithiasis. These limitations will be addressed in our future study.

In conclusion, we demonstrated that UTI patients with urolithiasis have a higher risk of developing uroseptic shock and AKI than those without urolithiasis. Therefore, careful medical history and imaging studies for urolithiasis are important for physicians to prevent and manage acute and severe complications in UTI patients.

## Data Availability Statement

The datasets generated for this study are available on request to the corresponding author.

## Ethics Statement

This study was conducted in concordance with institutional patient safety laws and has been approved by the Institutional Review Board of Chiayi Christian Hospital (approval no. CYCH-IRB-100015). This study was performed in accordance with the Declaration of Helsinki. The patients/participants provided their written informed consent to participate in this study.

## Author Contributions

All authors: participated in the interpretation of the studies and analysis of the data and also reviewed and approved the final version of the manuscript. C-YH: protocol/project development. C-YH and Y-YC: data collection or management. C-YH and T-HC: manuscript writing/editing. T-HC and Y-CL: data analysis. M-CH, P-HH, and M-CW: manuscript review. M-CW: scientific advisor.

### Conflict of Interest

The authors declare that the research was conducted in the absence of any commercial or financial relationships that could be construed as a potential conflict of interest.
